# Pathology and parasitology of free-ranging coyotes from Tennessee and South Carolina

**DOI:** 10.1371/journal.pone.0318645

**Published:** 2025-02-10

**Authors:** Eliza Baker, Michelle Dennis, Debra Miller, Chunlei Su, Alexa Rosypal von Dohlen, Mohamed A. Abouelkhair, Sarah A. Hamer, Alex Jensen, Richard Gerhold

**Affiliations:** 1 Department of Biomedical and Diagnostic Sciences, University of Tennessee College of Veterinary Medicine, Knoxville, Tennessee, United States of America; 2 Center for Wildlife Health, School of Natural Resources, University of Tennessee, Knoxville, Tennessee, United States of America; 3 One Health Initiative, University of Tennessee, Knoxville, Tennessee, United States of America; 4 Department of Microbiology, University of Tennessee, Knoxville, Tennessee, United States of America; 5 Department of Natural and Behavioral Sciences, Johnson C. Smith University, Charlotte, North Carolina, United States of America; 6 Department of Diagnostic Medicine, and pathobiology, Rowan University Shreiber School of Veterinary Medicine, Glassboro, New Jersey, United States of America; 7 Department of Veterinary Integrative Biosciences, Texas A&M University, College Station, Texas, United States of America; 8 Department of Forestry and Environmental Conservation, Clemson University, Clemson, South Carolina, United States of America; University of Massachusetts Amherst, UNITED STATES OF AMERICA

## Abstract

Coyotes are exposed to many parasites and pathogens of veterinary and zoonotic concern. To assess the prevalence of the diseases caused by these microbes, we opportunistically obtained coyote samples from a variety of sources including a GPS collaring study, rabies testing facilities, wildlife resources agents, and road-side mortalities. We performed necropsies, serological testing, fecal flotations, and molecular analyses on coyotes from Tennessee and South Carolina. *Dirofilaria immitis* (heartworm) infected 46% (41/89) of coyotes and was associated with eosinophilic alveolitis and arteritis. *Paragonimus kellicotti,* a zoonotic lung fluke, was found in 24% (17/71) of Tennessee coyotes, including one coyote with extrapulmonary infection affecting the liver and lymph nodes. *Trichinella* spp., a zoonotic nematode, was present in 17% (12/71) of Tennessee coyotes but was not associated with muscular inflammation. *Sarcoptes scabiei,* the causative agent of sarcoptic mange, was detected in one Tennessee coyote. Most coyotes (86% [90/105]) were seropositive for *Toxoplasma gondii,* while 8.5% (9/106) were seropositive for *Trypanosoma cruzi,* an emerging zoonotic, vector-borne parasite. This study demonstrated that coyotes are commonly exposed to numerous parasites and pathogens that affect people and pets and are excellent sentinels for these diseases.

## Background

Coyotes (*Canis latrans)* have expanded their range across the eastern United States in the last century [1]. Though their historic range was limited to the arid deserts and plains of the mid-West, the extirpation of predators like wolves (*Canis lupus*) and mountain lions (*Puma concolor*) as well as changes to the landscape have allowed coyotes to thrive in areas previously devoid of their presence [2]. Their generalist and adaptable nature has allowed them to colonize almost the entirety of North America. Unlike wolves and mountain lions, coyotes can thrive in suburban and fragmented habitats, and packs have been found in areas as dense as New York City and Chicago [3,4].

The impact of coyotes on the disease ecology of eastern North America is not yet clear. They can carry numerous pathogens of veterinary, human, and wildlife health importance, and changes in pathogen prevalence are possible as they interact with their new habitat. A baseline health assessment can indicate the range and prevalence of pathogens of concern. These data can be used by veterinarians and physicians to understand the risk to their patients, as well as by wildlife biologists to interpret diagnostic investigations and better understand the potential impacts of coyotes on eastern ecosystems.

There is some preliminary knowledge from the southeast. A survey in South Carolina (SC) found 35% (12/34) of coyotes had *Dirofilaria immitis* microfilariae in blood smears, and a 15% seroprevalence to canine distemper virus (CDV) (3/20) [5]. PCR testing for pathogens was not performed in that study. A study in Georgia tested for antibodies to several more pathogens including *Trypanosoma cruzi* and *Toxoplasma gondii,* finding a low prevalence (7% [2/27]) of the former and a high prevalence (92% [22/24]) of the latter [6]. Carcasses were available in that study, and 52% (16/31) of coyotes had adult *D. immitis* present in the heart or pulmonary arteries. Finally, a survey in North Carolina found a 47% (15/32) prevalence of *D. immitis* and a 17% (5/30) seroprevalence for CDV [7]*.*

Understanding the prevalence of these pathogens is important for both public health and animal management. *Dirofilaria immitis* is the causative agent of canine heartworm disease, one of the most common causes of cardiopulmonary disease in domestic dogs and a potential cause of respiratory disease and sudden death in domestic cats [8]. Spread by mosquitos, it is found in all fifty states, though prevalence is highest in the South [9]. CDV, similarly, is a cause of neurologic and respiratory disease in both domestic dogs and wildlife. It can affect a wide variety of species, causing epizootics in animals ranging from lions to seals to foxes [10]. *Toxoplasma gondii* is considered one of the most successful parasites of humans, infecting an estimated one-third of the global population [11]. Though most infections are mild or asymptomatic, infection in an immunocompromised or pregnant person can lead to severe disease or fetal abnormalities [11]. Animals, similar to people, can be asymptomatic or experience a variety of clinical signs affecting numerous organs including the central nervous system, lungs, eyes [12]. Another parasite that can affect both wildlife and domestic animals is *Sarcoptes scabiei,* a mite capable of causing severe pruritic dermatitis in domestic animals, wildlife, and people [13]. Epizootics can have significant, local population-level effects, with one epizootic in coyotes causing over 70% mortality, though these population declines are often temporary [14,15]. Finally, *T. cruzi,* the causative agent of Chagas disease, is a protozoan parasite that causes cardiac and gastrointestinal disease in humans, dogs, and other mammals in the Americas where triatomine insect vectors occur [16]. There is increasing awareness for locally-acquired human and canine Chagas disease in the southern United States [17,18].

Previous studies demonstrate that coyotes are exposed to various pathogens and parasites of concern, but their sample size was limited, and serology was the primary diagnostic tool. Though serology can provide an excellent baseline, there are multiple infectious and non-infectious processes that can be discerned in greater understanding by histopathology or molecular diagnostics. Coyotes can make excellent sentinels for a variety of diseases that affect people, pets, wildlife, and livestock. However, an accurate baseline is needed to maximize the utility of future health assessments. This study sought to fill in the knowledge gaps on infectious diseases and pathology of eastern coyotes.

## Materials and methods

### Sample collection and necropsies

**Fig 1:**
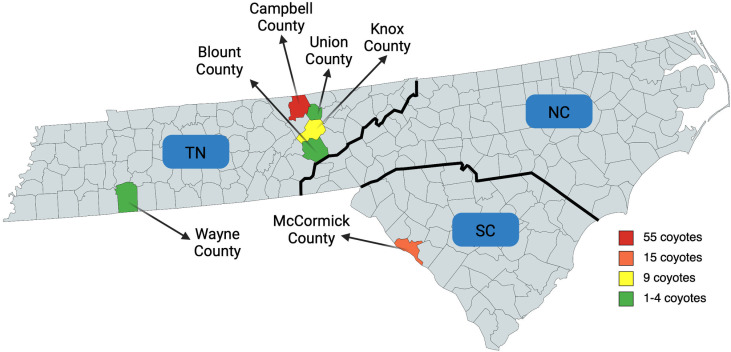
Map of counties sampled. The total number of coyote (*Canis latrans*) carcasses obtained from each county in Tennessee (TN) and South Carolina (SC) United States. Additional, antemortem serum, fecal, and/or whole blood samples were collected from 80 coyotes in McCormick County, SC. Created with MapCharts.

Samples were collected from a variety of sources within multiple southeastern states (Fig 1, S1 Table). A collaborator sent excess whole blood (n = 56), serum (n = 41), and fecal samples (n = 80) collected from a GPS collaring study of coyotes in South Carolina (SC) [19]. These samples were collected antemortem at the initial capture event. Any study animals that died while still collared were collected and saved frozen until necropsies could be performed (n = 15). Whole carcasses from eastern Tennessee (TN) were collected opportunistically from rabies testing facilities, roadside mortalities, and wildlife resources agents (n = 71). In addition, heart alone was collected from three TN coyotes. All capture and handling procedures of South Carolina coyotes were permitted by Clemson University IACUC permit no. AUP 2018-031 and USDA Forest Service permit no. USFS 2018-031. Samples from Tennessee were all collected opportunistically post-mortem, and no coyotes were killed for the purposes of this study.

Necropsies were performed as soon as possible on fresh carcasses, though those from SC and from Animal and Plant Health Inspection Service’s (APHIS) rabies testing facilities were frozen until necropsies could be performed (n = 21). Life stage (juvenile or adult) was determined based on tooth development and wear, body size, and uterine development in females [20]. Body condition score was determined on a 1–9 scale as described for dogs, with a BCS of 4–5 considered ideal [21]. Stomach contents were visually assessed and delineated into categories (vegetation, mammalian, avian, or human-associated) when the contents were grossly identifiable.

Heartworms were collected and saved in 70% ethanol. All worms were counted and sexed based on their size and tail morphology. Blood or blood clots were collected from the heart as soon as possible after death. An aliquot of the blood was centrifuged at 3000 rpm for 15 minutes to isolate serum, though this was not performed in previously frozen coyotes. Cerebrum, heart, lung, liver, and kidney were saved frozen. Samples from all major organs were collected and saved in 10% neutral buffered formalin, then trimmed and routinely processed for histology. Slides with suspected pathology were reviewed with a pathologist (authors DM and MD).

When encephalitis was present on histology, slides were sent to the University of Georgia and tested with immunohistochemistry (IHC) for canine distemper virus (CDV), West Nile Virus (WNV), and Eastern Equine Encephalitis virus (EEE).

### Serology

Because blood was collected post-mortem, serum was hemolyzed. Therefore, tests that are strongly affected by hemolysis were not run. *Trypanosoma cruzi* was assessed following the protocol described in the nation-wide working dog survey [18]. In short, we screened serum (n = 106) for antibodies using the commercially available Chagas Stat-Pak (ChemBio, Medford, NY). Positive samples were then assessed with two additional tests, when enough serum remained: the InBios Chagas Detect^TM^ Plus Rapid Test (InBios, Seattle, WA) and an indirect fluorescent antibody (IFA) test at the Texas A&M Veterinary Medical Diagnostic Laboratory. Both rapid tests are validated for use in humans, and the IFA test was validated for dogs. The use of these tests was off label for coyotes. Samples were considered true positives if they were positive on at least two independent tests*.* Modified agglutination tests (MAT) for *Toxoplasma gondii* antibodies were performed in the University of Tennessee Microbiology Laboratory (n = 105) as previously described, starting at a 1:25 dilution [22].

### Molecular analysis

DNA was extracted from 200µl whole blood (n = 130) and 25mg heart (n = 77) using Qiagen DNeasy Blood and Tissue kits (Qiagen Inc, Germantown, Maryland, USA) following manufacturer’s instructions. If *Trichinella* spp. larvae were seen on histology, DNA was extracted from paraffin-embedded muscle tissues. Paraffin was removed with an overnight incubation of xylene at 37°C followed by a second, thirty-minute incubation with fresh xylene. Remaining products after centrifugation and discarding of supernatant were washed with ethanol before beginning tissue lysis with buffer ATL and proteinase K. Manufacturer’s protocol was then followed apart from using a 2:1 deionized water to buffer AE ratio for the elution step. Multiplex PCR, to determine species of *Trichinella*, was performed following previous protocols but was not successful, potentially due to the poor DNA quality of formalin-fixed tissue and the decreased sensitivity of tissue extractions compared to individual larvae extraction [23]. Therefore, we performed PCR targeting a small segment of large ribosomal subunit as previously described which can confirm genus identity but not species [23].

DNA samples from blood and heart were tested for *T. cruzi* using primers that target the 24Sα DNA gene of trypanosomatids [24]. In addition, extracted samples were subjected to quantitative PCR using Cruzi 1/2/3 primers for detection of *T. cruzi* DNA [25]. A positive control of DNA extracted from *T. cruzi* Sylvio-X10 CL4 (ATCC 50,800, American Type Culture Collection [ATCC], Manassas, VA) was included in each reaction. Any samples with CT values < 35 were considered positive.

When unidentified trematodes were present, the DNA from individual trematodes was extracted with Qiagen DNEasy kits, and extracted DNA was tested using universal trematode primers targeting the 18S gene as previously described [26]. Skin scrapes were performed on all coyotes with crusts, alopecia, or dermatitis. Following morphological identification of mites, the skin scraped material was placed in a sterile microcentrifuge tube and DNA extracted using DNEasy kits. PCR was performed on extracted DNA to amplify the mitochondrial 16S rDNA of the identified mite species (i.e., *Sarcoptes scabiei*) as previously described [27].

All conventional PCR products were run on a 1.5% agarose gel, and PCR products with the correct base pair size were purified and sequenced with Sanger sequencing using a commercial facility (Eurofins Genomics, Louisville, KY). Bi-directional sequences were aligned and visualized using Sequencher (Ann Arbor, MI). Generated consensus sequences > 200 bp were submitted to GenBank, and accession numbers are shown in Table 1.

**Table 1:** PCR Protocols Primers used for nested or traditional PCR on coyote blood, tissue, or ticks are shown. Sequences > 200 bp were deposited in GenBank, and their accession numbers are shown in the GenBank column.

Organism	Gene	Sequence	Annealing temperature (°C)	PCR product (bp)	Citation	GenBank Accession Number
*Trypanosoma cruzi*	24s αRNA	D75: GCAGATCTTGGTTGGCGTAG D76: GGTTCTCTGTTGCCCCTTTT	59	262	Souto et al. 1999 [28]	NA, all negative
*Trichinella*	LRS rRNA ESV	NeF: TCTTGGTGGTAGTAGC NeR: GCGATTGAGTTGAACGC	55	~225	Zarlenga et al. 1999 [23]	PP544891 PP544892
Taeniidae spp.	COI	EchinoCOIF: TTTTTTGGGCATCCTGAGGTTTAT EchinoCOIR: TAAAGAAAGAACATAATGAAAATG	55	~450	Gasser et al. 1999 [29]	PP555939-PP555945
Trematodes	18S	Trem18SF: ATGGCTCATTAAATCAGCTAT, Trem18SR: TGCTTTGAGCACTCAAATTTG	60	~720	Diaz et al. 2022 [26]	PP544396- PP544398
*Sarcoptes scabiei*	16S	SSUDF (5′-GGGTCTTTTTGTCTTGGAATAAA-3′) SSUDR (5′-CTAAGGTAGCGAAATCATTAGC-3′)	60	135	Angelone-Alasaad et al. 2015 [27]	NA, only 135 bp

RNA was extracted from all lung samples (n = 76) using RNeasy kits from Qiagen following manufacturer’s instructions. Extracted RNA was tested in duplicate via reverse-transcriptase qPCR for canine distemper virus following the standard protocols of UTCVM’s Virology laboratory as previously described [30].

### Fecal flotation

Fecal flotation with centrifugation was performed on all samples using Sheather’s sugar solution as previously described [31]. Fecal samples with Taeniidae eggs seen on flotation were tested to differentiate between *Echinococcus* and *Taenia* infection. The contents of the flotation slide were transferred to a sterile microcentrifuge tube and put through two freeze-thaw cycles to aid in breaking the helminth eggshells. Samples were then extracted with Qiagen DNEasy kits following manufacturer’s instructions and tested with primers targeting the COI gene as previously described [29].

### Data analysis

Statistical analysis was performed in RStudio. Wilson’s 95% confidence intervals, a variation of Wald confidence intervals derived from data with two additional successes and failures which has better accuracy for small sample sizes, were calculated for all prevalences. The association between heartworm status and the presence of dermatitis, eosinophilic alveolitis, and/or endarteritis was assessed using Fisher’s exact tests. The association between *Trichinella* larvae and myositis was also assessed with a Fisher’s exact test. Chi-squared tests were used to determine differences in pathogen prevalence between states. The impact of human-associated diet items on body condition score was assessed using a Chi-squared test. The significance level was set to 0.05.

## Results

### Necropsy

A total of 86 coyote carcasses were collected and necropsied between 2018 and 2023, 71 from TN and 15 from SC (Fig 1, Table 2). In addition, heart alone was available from three TN coyotes, and whole blood (n = 56), serum (n = 41), and fecal (n = 80) samples were collected antemortem from SC coyotes. Estimated post-mortem interval ranged from a few hours to over two days. All but two (13/15) SC coyotes were female, while 36 TN coyotes were female and 35 were male. Most coyotes were young adults (12-24mo), though 16% (14/86) were estimated between 6-12 months old.

**Table 2:** Histopathologic lesions found in coyotes. Percentage positive is shown, with total number positive and total number assessed in parenthesis. 95% confidence intervals are shown in brackets. Some tissues have lower sample size either due to autolysis or because certain tissues (pancreas, bladder, and skin) were not routinely sampled until later in the study.

Category	Lesion	TN	SC	Total
Musculoskeletal	Chronic fracture	2.8% [0.2–10.4%]; (2/71)	13.3% [2.7–39.4%]; (2/15)	4.7% [1.5–11.8%]; (4/86)
	Myositis	14.1% [7.7–27.5%]; (10/71)	26.7% [10.7–52.4%]; (4/15)	16.3% [9.9–25.7%]; (14/86)
	Glossitis	12.2% [5.4–24.7%]; (6/49)	9.1% [0–40.2%]; (1/11)	11.7% [5.5–22.5%]; (7/60)
	*Trichinella*	16.9% [9.8–27.5%]; (12/71)	0% (0/15)	14.0% [8.1–23.0%]; (12/86)
Cardiac	Myocarditis	4.1% [1.0–12.0%]; (3/73)	33.3% [15.2–58.5%]; (5/15)	9.1% [4.5–17.2%]; (8/88)
	*Dirofilaria immitis*	44.6% [33.8–55.9%]; (33/74)	53.3% [30.2–75.1%]; (8/15)	46.1% [36.1–56.4%]; (41/89)
Pulmonary	*Paragonimus* *kellicotti*	23.9% [15.5–35.2%]; (17/71)	0% (0/15)	19.8% [12.7–29.5%]; (17/86)
	Eosinophilic Alveolitis	8.5% [3.7–17.7%]; (6/71)	26.7% [10.7–52.5%]; (4/15)	11.6% [6.3–20.4%]; (10/86)
	Endarteritis or periarteritis	8.5% [3.7–17.7%]; (6/71)	0% (0/15)	7.0% [3.0–14.8%]; (6/86)
	Other Pneumonia	11.3% [5.6–21.0%]; (8/71)	26.7% [10.7–52.5%]; (4/15)	14.0% [8.1–23.0%]; (12/86)
Urogenital	Interstitial nephritis	26.7% [17.1–39.1%]; (16/60)	42.9% [21.5–67.4%]; (6/14)	29.7% [20.5–41.0%]; (22/74)
	Renal abscesses	1.7% [0.1–10.1%]; (1/60)	0% (0/15)	1.3% [0–8.0%]; (1/75)
	Chronic renal infarction	6.7% [2.2–16.5%]; (4/60)	6.7% [0–32.2%]; (1/15)	6.7% [2.6–15.1%]; (5/75)
	Cystitis	15.1% [7.7–27.4]; (8/53)	0% (0/2)	14.5% [7.4–26.5%]; (8/55)
Skin	Dermatitis	29.0% [16.0–46.8%]; (9/31)	14.3% [0.1–53.6%]; (1/7)	26.3% [14.9–42.2%]; (10/38)
	Vasculitis	3.2% [0–17.8%]; (1/31)	0% (0/7)	2.6% [0–14.9%]; (1/38)
	Sarcoptic mange	1.4% [0–8.4%]; (1/71)	0% (0/15)	1.2% [0–7.0%]; (1/86)
Misc.	Pancreatic duct hyperplasia	43.8% [23.2–66.8]; (7/16)	0% (0/3)	36.8% [19.2–59.1%]; (7/19)
	Hepatitis	34.8% [18.8–55.2]; (8/23)	20.0% [2.5–64.1%]; (1/5)	32.1% [17.9–50.8]; (9/28)
	Sepsis	1.4% [0–8.4%]; (1/71)	6.7% [0–32.2%]; (1/15)	2.3% [0.2–8.7%]; (2/86)

**Fig 2:**
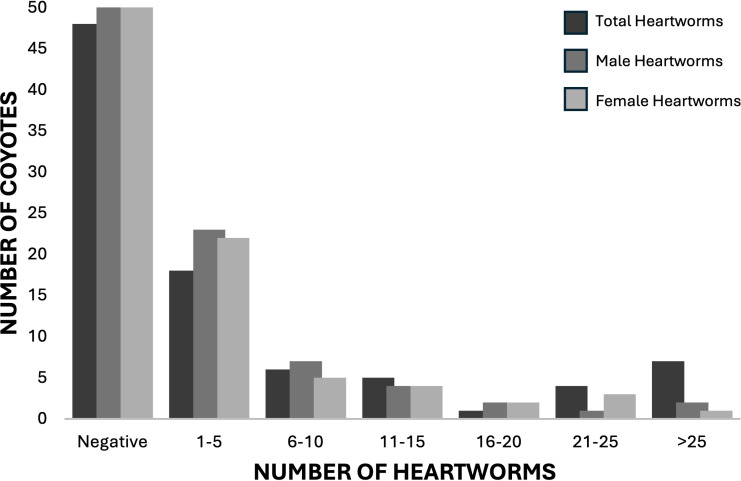
*Dirofilaria immitis* intensity found in coyotes (*Canis latrans*) from the Tennessee and South Carolina, United States.

*Dirofilaria immitis* were found in 53% (8/15) of SC coyotes and 44.6% (33/74) TN coyotes (Fig 2). The median number of adult *D. immitis* in positive coyotes was 7 (range: 1-109). Male and female *D. immitis* were found equally with no significant difference between the sexes (*X*^2^ = 0.11, df = 1, p = 0.73). Unisex infections were present in six coyotes - two male-only and four female-only infections.

Many human-associated items including pieces of stuffed animals, metal gears, plastic pieces, compost, and gravel were identified in coyote stomachs. These human-associated items were found in 5.6% (4/71) of TN and 14.3% (2/14) of SC coyotes. The presence of human-associated items was not significantly associated with a lower body condition score (F = 0.29, p = 0.59, df = 1).

### Histopathology

Histopathology of some organs, particularly brain, liver, pancreas, and kidney, was limited due to autolysis or tissue damage from euthanasia method in certain cases (Table 2). Some organs, like skin, tongue, bladder, and pancreas were not routinely collected until the latter half of the study. Finally, some organs were rendered unusable from gunshot wounds or injuries from automobiles.

#### Pulmonary.

**Fig 3:**
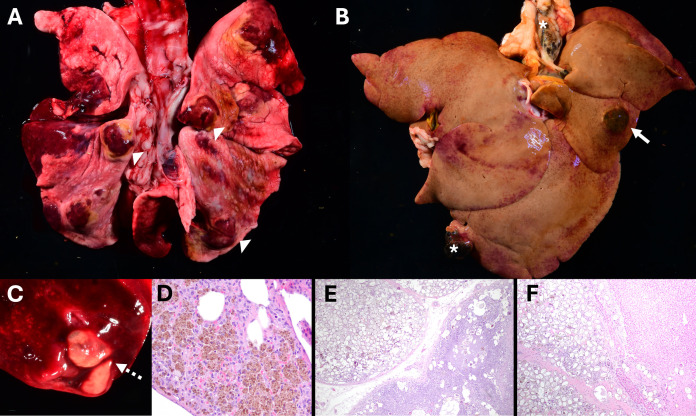
Extrapulmonary *Paragonimus kellicotti* infection in a Tennessee (USA) coyote (*Canis latrans*). A: Lung with multifocal hemorrhage and numerous firm cysts (arrow heads). B: Liver with large nodule (arrow) containing two adult *P. kellicotti* flukes and granular, black debris (parasitic hematin). Numerous smaller nodules were present in the lymph nodes, fat, and edges of the liver (asterisks). C: On cut surface, two flukes consistent with *P. kellicotti* (dotted arrow) were present within the center of each lung nodule. D: Pulmonary hemosiderosis in a coyote infected with *P. kellicotti.* Hemosiderin-laden macrophages were present throughout the pulmonary interstitium of coyotes with paragonimiasis. E: *Paragonimus kellicotti* eggs within a mesenteric lymph node. F: The liver nodule consisted of numerous *P. kellicotti* eggs with minimal inflammation. Some eggs expand past the fibrous wall of the nodule and into the hepatic parenchyma itself.

*Paragonimus kellicotti* flukes were encysted in the lungs of 24% (17/71) TN coyotes and were not found in the lungs of SC coyotes. Each cyst generally contained two adult trematodes. There was one severe infection with multiple pulmonary cysts and extrapulmonary involvement with several firm masses multifocally within the liver (Fig 3), as well as the perihepatic fat and lymph nodes. The largest liver nodule was 4 cm × 3 cm and contained two adult flukes. Histology revealed the liver and lymph node masses consisted almost entirely of *P. kellicotti* eggs (Fig 3E and 3F). PCR was performed on a section of the largest liver mass as well as an adult fluke from the lung to confirm species, and sequences were identical to each other and 99.7% identical to the only *P. kellicotti* 18S sequence in GenBank (HQ900670). Moderate to severe multifocal granulomatous and eosinophilic pneumonia affected all coyotes with *P. kellicotti* flukes. Histologic pulmonary changes consisted of egg masses and severe granulomatous inflammation surrounding the cysts. Hemosiderin-laden macrophages, sometimes far removed from the egg masses themselves, were present in the pulmonary interstitium of 88% (15/17) of *Paragonimus* infected coyotes (Fig 3D).

**Fig 4:**
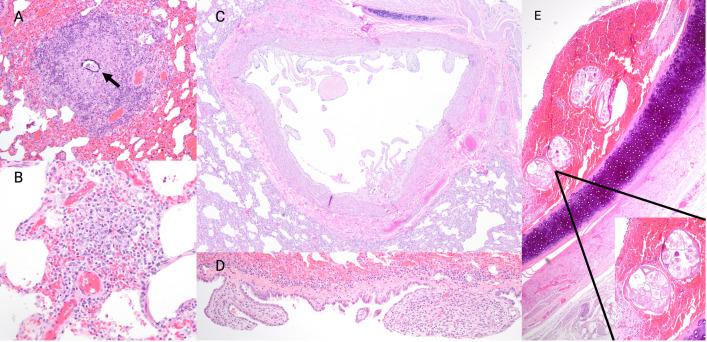
Parasitic respiratory lesions in coyotes (*Canis latrans*) from Tennessee and South Carolina, USA. A: Pulmonary granuloma in coyote with central mineralization (arrow), putatively associated with parasite migration. B: Mild eosinophilic alveolitis in a coyote with *Dirofilaria immitis* infection*.* Multifocal clusters of mixed inflammation consisting primarily of eosinophils with fewer macrophages and neutrophils expand the pulmonary interstitium. C: Villous endarteritis in a coyote presumably due to *D. immitis* infection. D: Mesothelial hyperplasia in a coyote likely secondary to pleural effusion associated with *Paragonimus kellicotti* infection. E: Focal hemorrhagic tracheitis associated with nematodes consistent with *Oslerus osleri* in a coyote. Mild mixed inflammation is present within the hemorrhagic nodule, and the tracheal epithelium is multifocally eroded.

Other parasite-associated changes included two coyotes with dystrophic mineralization at the center of focal granulomas, putatively associated with parasite migration (Fig 4A). Mild to moderate, multifocal, eosinophilic alveolitis was documented in 10 coyotes, with a significant association with *D. immitis* infection (Fisher’s exact test, p = 0.04, Fig 4B). Six TN coyotes had evidence of villous endarteritis or periarteritis, all of which were *D. immitis* positive (Fig 4C). One coyote had severe, granulomatous pneumonia due to *Angiostrongylus vasorum* infection, which we describe elsewhere [32].

Aside from parasite-associated pathology, lung lesions were uncommon. Diffuse bronchopneumonia was documented in one TN coyote, a large pulmonary abscess with intralesional cocci was present in one SC coyote, and embolic pneumonia was found in two septic coyotes, one from each state. Mild to moderate bronchopneumonia, sometimes associated with PCR-positivity for canine distemper virus, was present in six coyotes, though no inclusion bodies were noted. One coyote had villous mesothelial proliferation (Fig 4D) presumptively due to pleural effusion caused by *P. kellicotti* fluke migration. A second coyote had a focal mesothelial hyperplasia without a clear cause of chronic inflammation. Finally, one coyote had small mucosal nodules at the tracheal bifurcation containing nematodes consistent with *Oserlus osleri* (Fig 4E).

#### Dermatologic.

**Fig 5:**
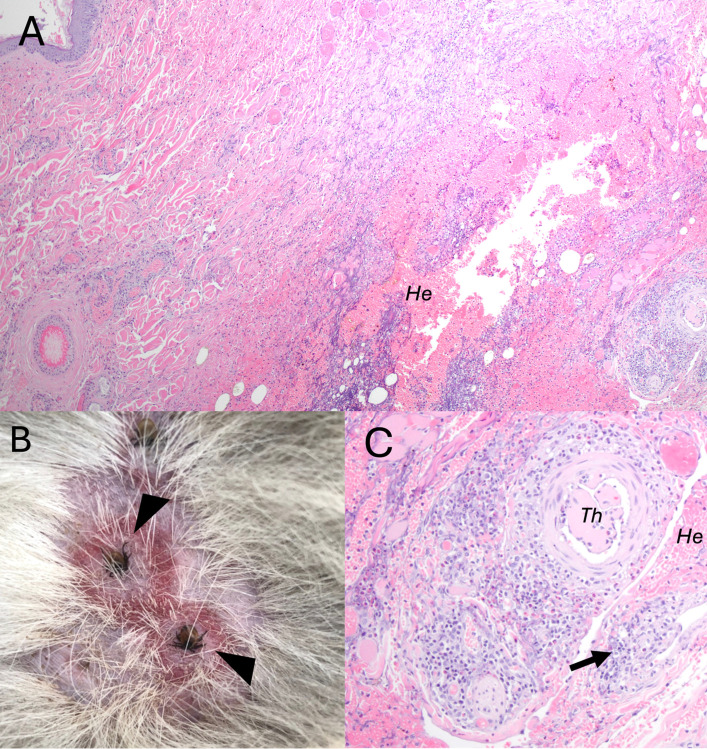
Tick-associated lesions in coyotes *(Canis latrans*) from Tennessee and South Carolina, USA. A: Photomicrograph of severe, acute cutaneous vasculitis associated with tick bites in *C. latrans.* Diffuse hemorrhage (*He*) with mixed inflammation and rare thrombi expand the subcutis. The vasculitis is constrained to a circular area surrounding the tick bites. B: Rings of erythema surround embedded adult *Ixodes scapularis* ticks (arrowheads) on a Tennessee coyote. C: Higher magnification view of the focal cutaneous vasculitis in A. An arterial thrombus (*Th*) is present, hemorrhage *(He)* expands the subcutis, and lymphocytes and eosinophils obscure the vessel walls of small arteries (arrow).

**Fig 6:**
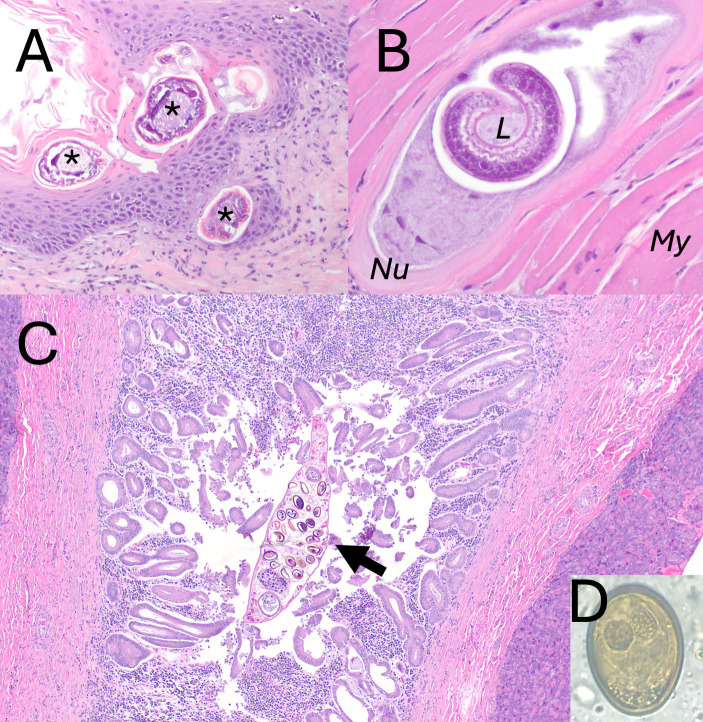
Parasitic lesions in coyotes *(Canis latrans*) from Tennessee and South Carolina, USA. A: *Sarcoptes scabiei* (asterisks) dermatitis in *C. latrans*. B: Intramyocytic *Trichinella* sp. larvae (L) in *C. latrans.* There is minimal associated inflammation surrounding the nurse cell (*Nu*). Surrounding myocytes (*My*) are unaffected. C: Chronic proliferative pancreatic dochitis in *C. latrans* due to *Eurytrema* sp. infection. The duct is hypertrophied with significant mixed inflammation filling the lumen. An adult *Eurytrema* fluke is present within the pancreatic duct (arrow). D: *Eurytrema* spp. egg on fecal flotation from the coyote with dochitis shown in E.

One coyote had marked eosinophilic cutaneous vasculitis with thrombi and intradermal hemorrhage associated with a circular rash surrounding bites from two *I. scapularis* ticks (Fig 5). An emaciated coyote from Knox County, TN, had sarcoptic mange (Fig 6A). The coyote had scabbing, crusting, pyoderma, and alopecia throughout most of its skin and had numerous mites consistent with *S. scabiei* present on a skin scrape. Sequencing of the 16S rDNA produced a 132 bp sequence that was 100% similar to other *S. scabiei* sequences in GenBank (e.g., KY290803). On histopathology, this coyote had evidence of sepsis, with bacterial emboli and associated suppurative inflammation present in the heart, lung, and kidney. The stomach of this coyote was empty aside from a small amount of green-to-tan fluid. No other coyotes had evidence of mange.

Dermatitis was present in 24% (9/38) coyotes including both pyoderma and allergic skin disease. Pyoderma, characterized by neutrophilic crusts and neutrophilic inflammation in the superficial dermis, was present in 16% (6/38), including the coyote with sarcoptic mange. Several coyotes (13% [5/38]) had features of allergic skin disease on histopathology characterized by eosinophilic or lymphohistiocytic inflammation in the superficial perivascular dermis and occasionally accompanied by hyperkeratosis. There was no association between *D. immitis* status and allergic skin disease (Fisher’s exact test, p = 0.16).

#### Musculoskeletal.

*Trichinella* spp. larvae were found in the skeletal muscle, tongue, or diaphragm in 17% (12/71) TN coyotes (Fig 6B). No *Trichinella* spp. were found in SC coyotes. *Trichinella* spp. were most common in the tongue (80% of positive cases), followed by skeletal muscle (58% of positive cases), and diaphragm (50% of positive cases). Multiplex PCR on paraffin-extracted pieces of tissue was not successful. However, traditional PCR was successful in cases where fresh tissue was available (n = 3). Amplified sequences were 100% identical to each other and to *T. britovi* sequences found in GenBank (KU374854). However, the short sequence length and paucity of *T. murrelli* sequences in GenBank precluded species identification. Though myositis was found in some coyotes, it was not associated with *Trichinella* larvae (Fisher’s exact test, p = 0.10).

#### Urinary.

Kidneys from one SC coyote and 11 TN coyotes were too autolyzed to accurately evaluate and were not included in the assessment. Lymphoplasmacytic interstitial nephritis typical of canine chronic kidney disease affected 28% (16/60) of TN coyotes and 43% (6/14) of SC coyotes. One TN coyote had multifocal neutrophilic abscesses within the cortex and medulla. Two coyotes from TN had a renal chronic infarction, with lymphoplasmacytic inflammation and fibrosis consuming a wedge-shaped streak in the cortex and medulla. Finally, one SC coyote had necrosuppurative pyelonephritis consuming half of its left kidney, with large swaths of necrosis, suppurative inflammation, and mineralization, presumptively a consequence of hematogenous bacterial infection given concurrent evidence of sepsis throughout multiple other organs.

Bladder was analyzed in 55 coyotes, with mild to moderate neutrophilic or eosinophilic cystitis of unknown etiology in 15% (8/55). There were no bladder stones, tumors, bacterial, or parasitic infection identified on necropsy or histology.

#### Gastrointestinal.

Minimal gastrointestinal histopathology was assessed due to autolysis. In addition, all GI tracts from the duodenum to the colon were sent to a collaborator performing an *Echinococcus* survey on coyotes. However, pancreas (n = 19) and liver (n = 28) were assessed when autolysis was minimal. Seven (37%) coyotes had moderate to marked eosinophilic and proliferative dochitis. Three of these had *Eurytrema* spp. eggs or adults within the pancreatic duct (Fig 6C), and two had *Eurytrema* spp. eggs on fecal flotation (Fig 6D). Numerous (>50) *Eurytrema* spp. adults were found incidentally within the small intestine of one coyote during necropsy. DNA was extracted from one adult trematode and tested with universal trematode primers [26]. The 731 bp consensus sequence was 98% similar to *E. pancreaticum* sequences in GenBank (KY490004). There are no *E. procyonis* sequences in GenBank, so the species identity is not clear.

Hepatitis affected 32% (9/28) of coyotes. Most cases consisted of mild focal or multifocal eosinophilic inflammation, likely related to parasite migration. One coyote had a large hepatic abscess with no causative agent identified.

#### Miscellaneous.

One coyote had moderate lymphocytic encephalitis with perivascular cuffing surrounding several vessels in its cerebrum and cerebellum. No distemper-like inclusions were present, and IHC of the slides, performed at University of Georgia, was negative for CDV, EEE, and WNV. The only other histopathologic lesions in this coyote were mild lymphoplasmacytic myocarditis and interstitial nephritis. Several coyotes (9.1% [8/88]) had myocarditis, typically consisting of mild to moderate lymphoplasmacytic inflammation. In the SC coyotes, this was often associated with *Hepatozoon americanum* infection, which we describe in detail elsewhere [33]. Myocarditis was less common in TN and consisted of rare multifocal lymphoplasmacytic clusters of inflammation. No causative agent was determined.

### Serology and molecular diagnostics

#### Toxoplasma gondii.

Most coyotes (85% [90/105]) were seropositive for *T. gondii*. Seropositivity was higher in SC (92% [36/39]) than TN (82% [54/66]), though this difference was not statistically significant (*X*^2^ = 1.43, df = 1, p = 0.23). The average titer in both states was 1:200. Just over 7% of coyotes from both states had titers > 1:3200 (8/105, Supp. Table 1).

#### Trypanosoma cruzi.

In total, 19.8% (21/106) of the coyotes were seropositive for *T. cruzi* on the Chagas Stat-Pak. Of those, 9 were also positive on the InBios ICT test, and two Stat-Pak positive cases did not have enough remaining serum to test. Only one SC coyote was positive on IFA with a titer of 1:1280. Therefore, 8.5% (9/106) of samples were positive on at least two serologic tests. None of the 130 tested coyotes were PCR positive for *T. cruzi* with either conventional or qPCR testing of blood and heart (Table 3).

**Table 3:** Serology and PCR results from coyotes from Tennessee (TN) and South Carolina (SC). Total positives and total number tested are shown in parenthesis, and 95% confidence intervals are shown in brackets. The Chagas Stat-Pak was used as the primary screening tool for *Trypanosoma cruzi* in coyotes, and only coyotes positive on the Stat-Pak were tested with the InBios ICT or IFA at Texas A&M University. Coyotes were considered positive if they were positive on 2 or more tests. The Modified Agglutination Test (MAT) was used for *Toxoplasma gondii* serology testing.

	Canine Distemper Virus PCR	*Trypanosoma cruzi* PCR	*Toxoplasma gondii* MAT	*T. cruzi* serology			
				Stat-Pak	InBios ICT	IFA	Pos on ≥ 2 tests
Organ tested	Lung	Whole blood and heart	Serum	Serum	Serum	Serum	Serum
TN	7.9% [3.1–17.8%]; (5/63)	0% (0/74)	81.8% [70.6–89.4%]; (54/66)	18.2% [10.6–29.4%]; (12/66)	20% [4.9–52.2%]; (2/10)	0% (0/4)	3.0% [0.3–11.2]; (2/66)
SC	15.4% [3.4–43.7%]; (2/13)	0% (0/56)	92.3% [78.8–98.0]; (36/39)	22.5% [12.2–37.8%]; (9/40)	77.8% [44.1–94.3%]; (7/9)	11.1% [0.2–46.0%]; (1/9)	17.5% [8.5–32.3%]; (7/40)
Total	9.2% [4.3–18.2%]; (7/76)	0% (0/130)	85.7% [77.6–91.2%]; (90/105)	19.8% [13.3–28.5%]; (21/106)	47.4% [27.4–68.2%]; (9/19)	7.7% [0–35.8%]; (1/13)	8.5% [4.4–15.6%]; (9/106)

#### Canine distemper virus (CDV).

qPCR for distemper virus was positive in 9.2% (7/76) of the coyotes (Table 3). Only two coyotes were strongly positive (CT value of 14.9 and 20.8), while the rest had values between 30–35. Both strongly positive coyotes and two of the weakly positive coyotes had interstitial or bronchopneumonia, though no inclusion bodies were seen.

### Parasitology

Fecal flotations were performed on 133 coyotes (Table 4). Parasite eggs were found at much higher frequencies in the fresh (TN) compared to ethanol-preserved (SC) stool with 94% (49/52) of TN coyotes positive for at least one parasite on fecal float compared to only 36% (29/80) of SC coyotes. TN coyotes had the highest prevalence of *Ancylostoma* spp. (77%) followed by *Sarcocystis* spp. (54%) and capillarid species (29%). *Eurytrema* spp. eggs were found in 7.7% (4/52) TN coyotes (Fig 6D). Taeniidae spp. were found in 10% (8/80) SC coyotes and 7.7% (4/52) TN coyotes. Cestode PCR was successful in seven of these samples. Six samples were 92-98% similar to *T. pisiformis* sequences in GenBank, and one sample was 99.2% similar to *T. hydatigena* (Table 1).

**Table 4:** Fecal flotation results from coyotes from Tennessee (TN) and South Carolina (SC). The percentage of coyotes positive for each parasite is shown, with the total number positive and total number tested in parenthesis and 95% confidence intervals shown in brackets. The *Neospora-*like category includes both *N. caninum* and *Hammondia* spp. since they are indistinguishable on fecal flotation. Samples from SC were stored in ethanol for 1–6 months before processing, while samples from TN were processed fresh within 24 hours of collection. Parasites in the other category included *Uncinaria, Strongyloides,* and dorsal-spine larvae.

Species	TN	SC	Total
*Ancylostoma* *caninum*	76.9% [63.7–86.3%]; (40/52)	2.5% [0.2–9.3%]; (2/80)	31.8% [24.4–40.2%]; (42/132)
*Sarcocystis* spp.	53.8% [40.5–66.6%]; (28/52)	13.8% [7.7–23.2%]; (11/80)	29.5% [22.4–37.9%]; (39/132)
*Neospora-*like	21.2% [12.2–34.3%]; (11/52)	5.0% [1.6–12.7%]; (4/80)	11.4% [6.9–18.1%]; (15/132)
*Cystoisospora* spp.	9.6% [3.8–21.2%]; (5/52)	10.0% [5.0–18.8%]; (8/80)	9.8% [5.8–16.3%]; (13/132)
Capillarid spp.	28.8% [18.3–42.4%]; (15/52)	3.8% [0.9–11.0%]; (3/80)	13.6% [8.8–20.7%]; (18/132)
*Paragonimus kellicotti*	25.0% [15.2–38.3%]; (13/52)	2.5% [0.4–9.6%]; (2/80)	11.4% [6.9–18.1%]; (15/132)
*Trichuris*	17.3% [9.2–30.0%]; (9/52)	2.5% [0.2–9.3%]; (2/80)	8.3% [4.6–14.5%]; (11/132)
*Taeniidae*	7.7% [2.5–19.4%]; (4/52)	10.0% [5.0–18.8%]; (8/80)	9.1% [5.2–15.4%]; (12/132)
*Physaloptera*	7.7% [2.6–18.8%]; (4/52)	0% (0/80)	3.0% [1.0–7.9%]; (4/132)
*Toxocara*	15.4% [7.8–27.9%]; (8/52)	2.5% [0.2–9.3%]; (2/80)	7.6% [4.1–13.6%]; (10/132)
*Eurytrema*	7.7% [2.6–18.8%]; (4/52)	0% (0/80)	3.0% [1.0–7.9%]; (4/132)
Other	21.2% [12.2–34.3%]; (11/52)	0% (0/80)	8.3% [4.6–14.5%]; (11/132)

## Discussion

We detected numerous pathogens of human and veterinary importance in the sampled coyotes. For multiple pathogens of concern, coyotes had a higher prevalence than what has been documented in pets or people, making them excellent sentinels for these diseases [34,35].

### Necropsy and histopathology

#### Pulmonary.

The high prevalence of *P. kellicotti* in Tennessee*,* both in the fecal flotations (25%) and the lungs (23.9%), was unexpected. Previous analyses have found *P. kellicotti* in less than 4% of coyotes in Florida and Ontario [36,37]. Even in the primary definitive host, mink, prevalence is typically < 20% [35]. This study is only the second report of extrapulmonary *Paragonimus* in a canid. The previous report occurred in 1976 in a mixed breed dog with numerous *Paragonimus* cysts in its lungs, and egg granulomas found throughout its liver, spermatic cord, and mediastinal lymph nodes [38]. Though rare, our study further demonstrates that extrapulmonary involvement may occur in cases of high parasite-burden. Of the necropsy-positive coyotes that received fecal floatationss, 92% (12/13) had *Paragonimus* eggs on fecal flotation, demonstrating a high sensitivity of centrifugal fecal flotation for infection. The fecal-negative coyote only had two cysts present in the lungs.

Most TN samples came from one wildlife management area (WMA) in Campbell County, while all SC samples came from a single county. Therefore, though the prevalence of *Paragonimus,* and other pathogens found in this study, is likely comparable to neighboring counties, we cannot determine what trends may be related to the specific ecology of those regions. This is particularly salient for *Paragonimus,* which relies on both gastropod and crustacean hosts for its lifecycle and therefore has more unique ecologic requirements for parasite maintenance in an environment.

#### Musculoskeletal.

TN coyotes had a high prevalence of *Trichinella* spp. infection. We were unable to confirm species, likely since most genotyping protocols require digestion and assessment of individual larvae, which we did not attempt for this study. *Trichinella murrelli* is one of the most common species in wildlife in the temperate United States and has been documented in coyotes in the past [39,40]. The prevalence of 17% in TN coyotes is higher than most previous reports in coyotes, which typically range from 4-10% prevalence [39,41–43]. Only one study from Wisconsin found a higher prevalence of 26% (11/42) [40]. The last survey in TN in 1987 examined 170 coyote diaphragms and did not find any positives [44]. It is unclear from the description if they performed tissue digestion or squash preparations. Regardless, we report a higher prevalence than previously documented in TN, even though we did not perform tissue digestion, the gold standard for diagnosis. The underlying reason behind the differences in *Trichinella* prevalence between the two states is unclear, and further surveillance in a wider geographic range is justified. Though the zoonotic potential of *Trichinella* in coyotes is essentially minimal, as they are not game animals, they are excellent sentinels for the prevalence in an area due to their scavenging nature [45].

#### Miscellaneous.

There were several histopathologic findings without a clear underlying cause. We were unable to determine an etiology for the myocarditis in TN coyotes, the mild to moderate eosinophilic to neutrophilic cystitis in coyotes from both states, and the lymphocytic encephalitis in one TN coyote. The lesions consistent with allergic skin disease were often present in areas surrounding tick bites, though many coyotes were infested with ticks without these dermatologic changes. Therefore, the underlying cause of allergic skin disease in these coyotes remains unclear.

The presence of marked autolysis in some coyotes (n = 11) and freeze artifact in others (n = 16) limited the usefulness of histology in certain cases, particularly when evaluating gastrointestinal organs. Histopathology of brain, intestine, liver, and pancreas were often unusable due to these changes, limiting our assessment of some of the diseases of interest including CDV. In addition, using only post-mortem samples from Tennessee limited our ability to test serum, which kept us from comparing our results to the previous southeastern coyote surveys that assessed exposure to CPV, CDV, and West Nile Virus.

### Serology and molecular diagnostics

#### Trypanosoma.

No serologic tests for *T. cruzi* are validated in coyotes, and studies vary in their methodology for determining positives. We followed a conservative approach, considering a sample positive only if it was positive on two separate tests. Though 19.8% (21/106) of coyotes in our study were seropositive on the STAT-Pak alone, only 8.5% (9/106) were positive on both Stat-Pak and InBios. IFA testing was limited in our study due to the low volume and quality of serum. However, only one SC coyote was positive on IFA, showing a poor agreement between tests.

Prevalence in SC (17.5% [7/40]) was higher than prevalence in TN (3.1% (2/64), though not significantly so (Fisher’s exact test, p = 0.34). However, a higher prevalence in more southern counties is expected based on the known distribution of *T. cruzi* [46]. Few similar surveys exist with which to compare our findings. An SC survey tested two coyotes and 26 gray foxes with both the InBios ICT and IFA. Neither of the coyotes were positive, but 8% (2/26) of the foxes were positive on both tests, similar to our total prevalence [47]. The only previous coyote survey in TN used the InBios ICT exclusively and found a 9.5% (2/21) prevalence, again similar to our total prevalence although the use of only one serologic test would lead to a higher reported prevalence. The working dog survey, which our methods are based on, found a higher seropositivity in TN working dogs (11%) than we found in coyotes. This is surprising, as coyote exposure to triatomine bugs would theoretically be higher than owned dogs. This could be explained by the high incidence of *T. cruzi-*infected bugs in dog kennels or differences in prevalence between eastern TN and the rest of the state [48]. As concern for the spread of *T. cruzi* in the United States grows, wildlife surveillance will remain a vital component of assessing distribution and risk.

#### Canine distemper virus (CDV).

There are no comparable molecular studies assessing CDV prevalence in coyotes. Seroprevalence is variable, ranging between 10%-56% in previous studies across the United States [49–52]. A study of raccoons and foxes found a 74% (43/58) qPCR prevalence including in 55% (12/22) of clinically healthy animals [53]. Only 17 of the PCR-positive wildlife in that study had lung pathology, suggesting either an acute or carrier state for raccoons and foxes. Our study provides evidence that coyotes may also be capable of subclinical or post-clinical shedding, and that coyotes may be PCR positive without any histologic evidence of pathology.

#### Toxoplasma gondii.

A high prevalence (86%) of coyotes were seropositive for *T. gondii.* Most surveys have found *T. gondii* exposure in at least half of tested coyotes, though a survey in Colorado found only a 20% positivity (5/25) and a survey in Alaska found no seropositive coyotes (0/12) [54,55]. Prevalence was higher in other studies, with 51–61% of coyotes from Texas, Indiana, and Wisconsin seropositive, and 92% (22/24) of coyotes from Georgia seropositive for *T. gondii* [6,56,57]. Our results align most closely with that of Georgia, showing exposure to this zoonotic pathogen is very common in the southeastern United States. Coyote exposure is higher than many other mammals, with one study in the southeastern United States finding *T. gondii* seropositivity in 72% (16/22) coyotes compared to only 41% (99/241) white-tailed deer (*Odocoileus virginianus*), 50% (17/34) of raccoons (*Procyon lotor*), and 51% (51/100) feral hogs (*Sus scrofa*) [58]. Coyotes do not transmit *T. gondii* to people, but their scavenging nature places them into contact with many different potential sources of this pathogen, making them excellent sentinels, particularly in areas where pathogen exposure may be low.

### Fecal flotation

The presence of *Eurytrema* spp. was surprising, since many parasite surveys have been performed in coyotes with only one finding *Eurytrema* in a single coyote-red wolf hybrid from the western Gulf Coast [5,6,59–61]. Raccoons are considered the definitive host of *E. procyonis,* and *Eurytrema* has also been documented in domestic cats, foxes, and maned wolves [61–64]. It is worth noting that all but one *Eurytrema* spp. and *P. kellicotti* infection were from Campbell County, TN. It is possible that mollusk, grasshopper, or crayfish abundance is higher in this area, creating a hotspot for these trematode infections. However, further research with a greater sample size from other counties would be necessary to make that determination.

The high prevalence of *Ancylostoma, Sarcocystis,* and *Neospora*-like species is similar to what has been found in previous fecal flotation surveys in wild canids, though there is significant geographic variability [36,59]. Although *Echinococcus canadensis* has been reported in elk in TN, none of the coyotes from TN or SC were positive for this zoonotic parasite [65]. However, fecal flotation is not a sensitive method for diagnosing *Echinococcus* spp. The gold standard involves intestinal scraping and sieving to identify adults within the gastrointestinal tract. Gastrointestinal tracts from our study were sent to a collaborator performing a nation-wide *Echinococcus* surveillance study in coyotes, limiting assessment in this study.

Fecal flotation appeared to be impacted by the months-long storage times in ethanol, which has been shown to decrease fecal egg counts in experimental testing [62]. The results of fecal flotation indicated different infection rates of most of the parasites tested between TN and SC coyotes (Table 4). Therefore, the prevalence reported from SC should be viewed as minimum occurrence rather than true prevalence, and the results should not be compared with the results from TN, which were obtained with fresh feces. The difference in storage methods and duration makes comparison of histology and fecal results between the two states challenging and is a major limitation of this study.

## Conclusion

Coyotes’ abundance, generalist nature, and wide geographic range exposes them to many pathogens that affect domestic animals, wildlife, and people. From the discovery of pathogens emerging in the southeast, like *T. cruzi,* to determining prevalence data for parasites common in the southeast, like *D. immitis,* coyote surveillance can fill in many knowledge gaps on diseases of both veterinary and human health importance. The One Health approach, or a collaborative approach to health that prioritizes the connection between humans, animals, and the environment, has only proven more vital as wildlife and climate change-associated diseases continue to rise. Coyote surveillance is a useful component of this approach. We discovered high prevalence of pathogens of public health concern including *Trichinella* spp. and *T. gondii* as well as pathogens of primarily animal concern like *D. immitis* and canine distemper virus*.* Coyotes therefore serve as useful sentinels for pathogens across the One Health spectrum. Further surveillance in other areas of the United States may elucidate trends or changing risks for many of these pathogens.

## Supporting Information

S1 Table Samples collected and tests performed on coyotes from Tennessee and South Carolina, USA. (XLSX)
